# A genotype-phenotype correlation matrix for ABCA4 disease based on long-term prognostic outcomes

**DOI:** 10.1172/jci.insight.156154

**Published:** 2022-01-25

**Authors:** Winston Lee, Jana Zernant, Pei-Yin Su, Takayuki Nagasaki, Stephen H. Tsang, Rando Allikmets

**Affiliations:** 1 Deparment of Genetics and Development,; 2 Department of Ophthalmology, and; 3 Department of Pathology and Cell Biology, Columbia University Irving Medical Center, New York, New York, USA.

**Keywords:** Genetics, Ophthalmology, Genetic variation, Monogenic diseases, Retinopathy

## Abstract

**Background:**

More than 1500 variants in the ATP-binding cassette, sub-family A, member 4 (*ABCA4*), locus underlie a heterogeneous spectrum of retinal disorders ranging from aggressive childhood-onset chorioretinopathy to milder late-onset macular disease. Genotype-phenotype correlation studies have been limited in clinical applicability as patient cohorts are typically small and seldom capture the full natural history of individual genotypes. To overcome these limitations, we constructed a genotype-phenotype correlation matrix that provides quantifiable probabilities of long-term disease outcomes associated with specific ABCA4 genotypes from a large, age-restricted patient cohort.

**Methods:**

The study included 112 unrelated patients at least 50 years of age in whom 2 pathogenic variants were identified after sequencing of the *ABCA4* locus. Clinical characterization was performed using the results of best corrected visual acuity, retinal imaging, and full-field electroretinogram testing.

**Results:**

Four distinct prognostic groups were defined according to the spatial severity of disease features across the fundus. Recurring genotypes were observed in milder prognoses, including a newly defined class of rare hypomorphic alleles. PVS1 (predicted null) variants were enriched in the most severe prognoses; however, missense variants were present in a larger-than-expected fraction of these patients. Analysis of allele combinations and their respective prognostic severity showed that certain variants, such as p.(Gly1961Glu), and both rare and frequent hypomorphic alleles, were “clinically dominant” with respect to patient phenotypes irrespective of the allele in *trans*.

**Conclusion:**

These results provide much-needed structure to the complex genetic and clinical landscape of ABCA4 disease and add a tool to the clinical repertoire to quantitatively assess individual genotype-specific prognoses in patients.

**FUNDING:**

National Eye Institute, NIH, grants R01 EY028203, R01 EY028954, R01 EY029315, P30 19007 (Core Grant for Vision Research); the Foundation Fighting Blindness USA, grant no. PPA-1218-0751-COLU; and Research to Prevent Blindness.

## Graphical abstract

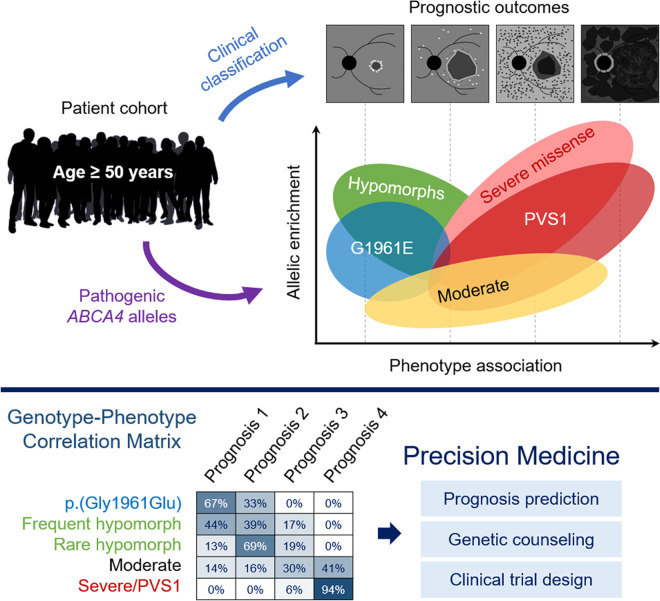

## Introduction

Pathogenic variants in the ATP-binding cassette, sub-family A, member 4 (*ABCA4*), gene are the underlying molecular cause of a large and complex group of autosomal-recessive retinal degenerative disorders characterized by progressive loss of central vision (1). The most well-known phenotype is the eponymous Stargardt disease (STGD1, MIM #248200; ref. 2); however, advances in genetic screening capabilities, aided by high-resolution diagnostic imaging technology, have broadened the phenotypic profile of ABCA4 disease to an expansive clinical spectrum encompassing severe adolescent-onset to mild late-onset retinal disorders (3). This phenotypic heterogeneity is matched by an equally extensive array of pathogenic variations across the approximately 140 kb–spanning *ABCA4* locus (1p22.1). To date, more than 1500 disease-causing variants have been identified in patients (4). Consistent with the model that clinical phenotypes are dependent on the residual activity of ABCA4 protein (5, 6), variants resulting in null alleles, such as stop-gain, frameshift, canonical splice site, and large copy number variants, have been documented in the most severe phenotypes, such as cone-rod dystrophy, rapid-onset chorioretinopathy (ROC), and even generalized choriocapillaris dystrophies with retinitis pigmentosa–like features (6–10).

More recently, the complex genetic architecture of milder ABCA4 disease manifestations has been uncovered. The most frequent pathogenic allele, c.5882G>A [p.(Gly1961Glu)], is associated with a slowly progressing disease trajectory in patients who often present with transient phenotypes, such as bull’s eye maculopathy and occult macular dystrophy (11, 12). Despite being highly prevalent in patients, the disease penetrance of this allele has been disputed as its frequency in the general population is also relatively high (minor allele frequency [MAF] ≈ 0.5% in Europeans) and in certain ethnic groups is much higher (13, 14). We recently resolved this controversy, for the most part, by showing that the contribution of an additional deep intronic variant, c.769-784C>T (15, 16), present in *cis*, is required for clinical penetrance, particularly in homozygotes (17). Alleles causing late-onset ABCA4 disease, such as c.5603A>T [p.(Asn1868Ile] and c.4253+43G>A, occur at even higher frequencies in the population of European descent (up to 7% MAF) and, unlike p.(Gly1961Glu) and other disease alleles, are only clinically penetrant under the condition that the allele in *trans* is sufficiently deleterious (18, 19).

Steady progress in defining genotype-phenotype correlations has been made, and the addition of such knowledge to the medical repertoire has inarguably elevated the clinical care of patients. Studies to date have often relied on cross-sectional cohorts of a patient population that include all age groups. As a result, the correlated “phenotype” studied is often a stage-specific feature, e.g., bull’s eye maculopathy or occult macular dystrophy. Such information, while no doubt useful at the diagnostic stage, is not informative of an individual patient’s long-term prognosis. To address this issue, we constructed a genotype-phenotype correlation matrix based on the most temporally advanced phenotypes of 112 patients aged 50 years or older who have 2 confirmed pathogenic variants in *ABCA4* coupled with comprehensive clinical characterization. We also reclassified many frequent disease-causing alleles, thereby further clarifying the impact of *ABCA4* variants on clinical outcome. Our findings provide structure to the complex genotype-phenotype correlation landscape of ABCA4 disease and establish a quantitative approach for predicting the prognosis of individual patients by clinicians and genetic counselors and for assessing the severity of pathogenic variants. The prognostic matrix will also aid in selecting specific patient groups for clinical trials, depending on the specific therapeutic application.

## Results

### Four clinically defined prognostic outcomes of ABCA4 disease.

**Figure 1:**
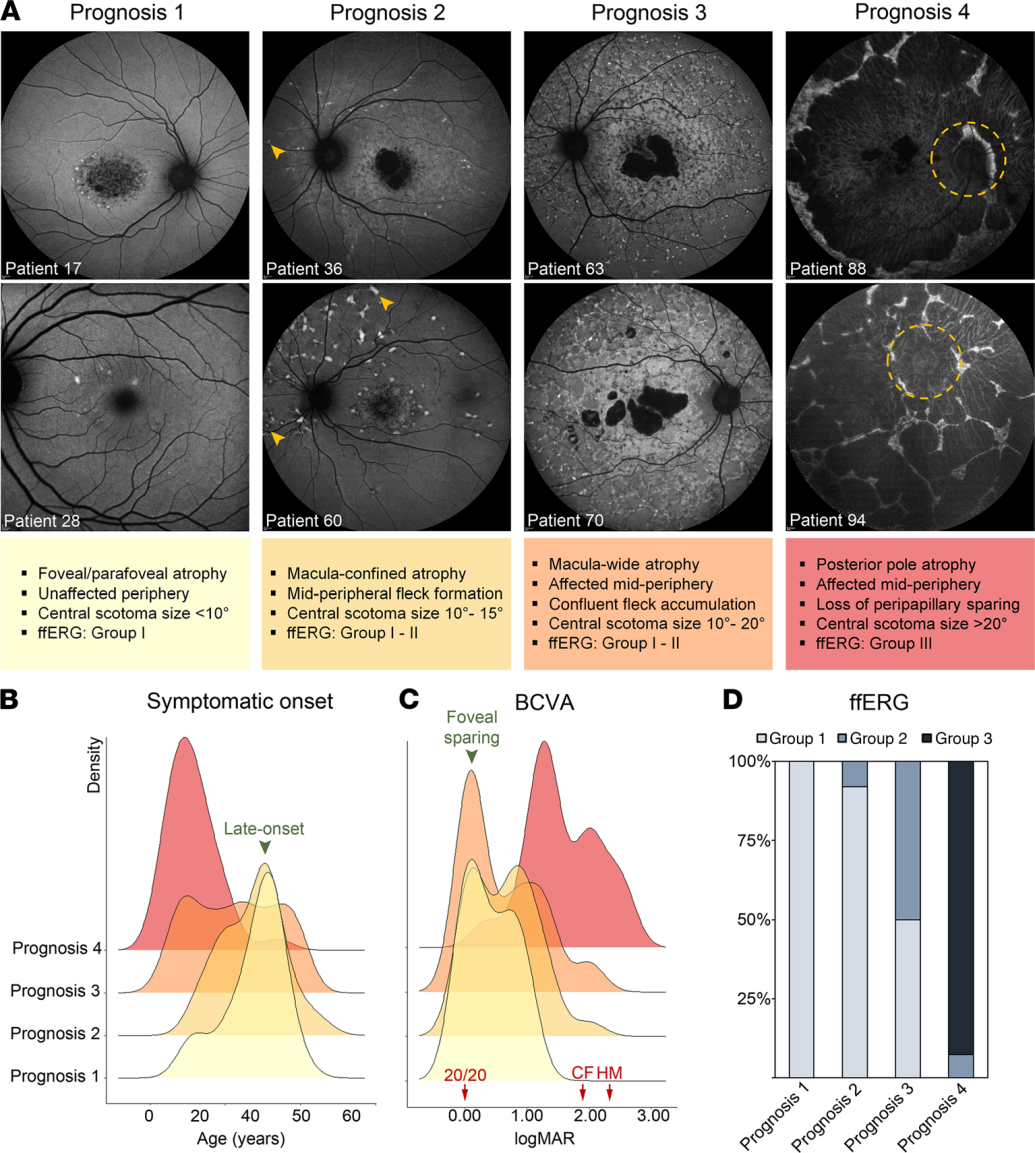
Clinical characteristics of 4 prognostic outcomes observed in 112 patients (≥50 years of age) with ABCA4 disease. Each prognosis was defined according to observable spatial progression of disease features detected at the most recent visit in each patient. (**A**) Representative autofluorescence images and clinical descriptions of patients in each prognosis classification. Extramacular development of flecks in prognosis 2 is indicated by yellow arrowheads. The position of the optic nerve is encircled by the dotted yellow line. The fields of view are 55° in all autofluorescence images except P28 (30°). (**B**) Ridgeline plots of the distribution of ages at which visual symptoms of all patients were first reported for each prognosis category (bandwidth = 5.83). (**C**) Density plots of the best-corrected visual acuity (BCVA) of the least-impaired eye of all patients (bandwidth = 0.246). BCVAs were presented in logMAR units with corresponding Snellen equivalents (20/20, counting fingers [CF] and hand motion [HM], red arrows) provided. (**D**) Proportion of ffERG groupings according to the classification by Lois et al. (37) for each prognosis category. ffERG, full-field electroretinogram; group 1, normal responses; group 2, attenuation of cone responses; group 3, attenuation of cone and rod responses; logMAR, logarithm of the minimum angle of resolution.

Demographic, clinical, and genetic characteristics of all 112 patients in the study are summarized in Supplemental Table 1; supplemental material available online with this article; https://doi.org/10.1172/jci.insight.156154DS1 Clinical data from the most recent visit for each patient were used in the study. Each patient was categorized into 1 of 4 prognosis categories based on the observable spatial progression of ABCA4-associated disease features in the fundus (Figure 1A) by age 50 years or older. Patients categorized as having prognosis 1 (*n* = 28) had the mildest disease outcome (in the cohort), manifesting early retinal pigment epithelium atrophy within the central macula without any apparent pisciform flecks. Patients with prognosis 2 (*n* = 31) were at a more progressed stage of chorioretinal atrophy across the macula and developed nascent flecks that appeared outside the vascular arcades (Figure 1A, yellow arrowheads). All patients with prognosis 3 (*n* = 20) had multifocal regions of chorioretinal atrophy, which, in some cases, extended beyond the macula and exhibited a pattern of highly confluent flecks in nonatrophic regions. Patients with prognosis 4 (*n* = 33) progressed to the stage characterized by the large atrophic, coalescing lesions across the entire posterior pole.

There were no significant differences in the mean age of patients between prognosis groups (Supplemental Data 1). The mean age of symptomatic onset was earliest among patients with prognosis 4 (17.1 years) compared with the milder prognostic groups, which had peak distributions at 41.7 years (prognosis 1) and 40.9 years (prognosis 2) due to the large number of late-onset disease cases in these latter groups (Figure 1B, see Supplemental Data 2). Best corrected visual acuity (BCVA) from the most recent visits was also poorest among patients with prognosis 4, of whom approximately 40% were assessed by counting fingers or by an assessment for even worse visual acuity (*P* < 0.00001; Figure 1C, see Supplemental Data 3). Comparatively, BCVA distributions were multimodal among patients with prognoses 1 to 3, most of whom had 20/200 (logMAR 1.00) or worse and 20/20 in cases with foveal sparing. Full-field electroretinogram responses were largely unremarkable in prognoses 1 and 2 (Figure 1D). Significant defects were found in prognosis 3 (50% Lois Group 2) and prognosis 4 (93% Lois Group 3, see Methods). There were no significant differences in mean age of patients across prognosis groups (*P* = 0.254) (Supplemental Data 1).

### Classification of p.(Gly1961Glu), p.(Asn1868Ile), and a potentially new class of rare hypomorphic alleles.

**Figure 2:**
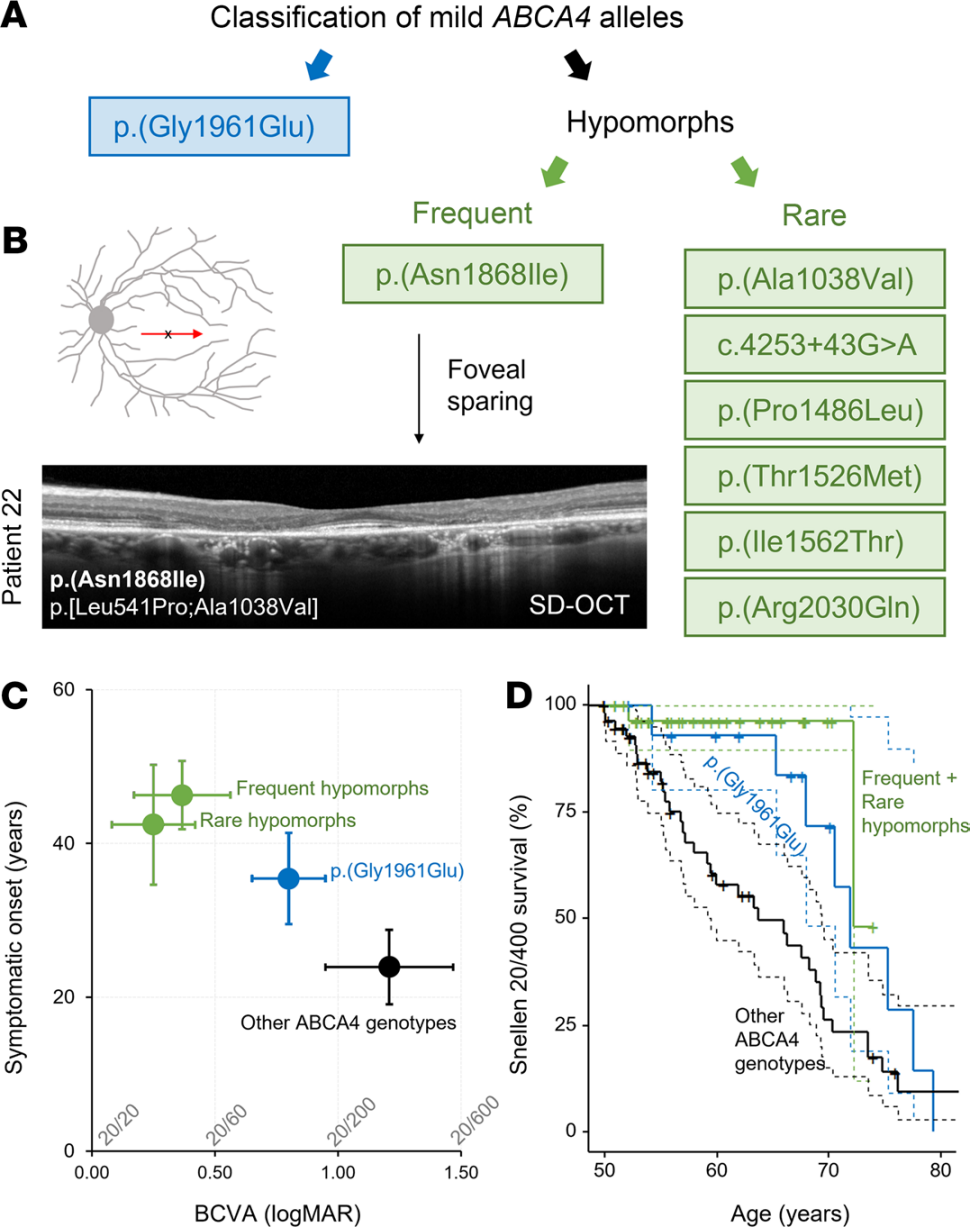
Classification and phenotypic characterization of mild *ABCA4* alleles. (**A**) Mild *ABCA4* alleles identified in patients with mild prognoses included p.(Gly1961Glu) and 2 hypomorphic allele subgroups: frequent hypomorphs, which consisted of p.(Asn1868Ile), and rare hypomorphs, which consisted of p.(A1038V), c.4253+43G>A, p.(Pro1486Leu), p.(Thr1526Met), p.(Ile1562Thr), and p.(Arg2030Gln). (**B**) Horizontal (6 mm) spectral-domain optical coherence tomography (SD-OCT) scan showing structural preservation of the fovea in patient 22, an allele-specific subphenotype common among p.(Asn1868Ile) genotypes. (**C**) Scatter plot of average age of onset (years) versus average best corrected visual acuity (BCVA) of the least deteriorated eye in patients within all patients/genotypes with p.(Gly1961Glu) (blue), frequent hypomorph p.(Asn1868Ile) (green), rare hypomorph (green), and all other allele combinations (black) in the study cohort. Horizontal and vertical bars represent ±95% confidence intervals. BCVAs are provided as logMAR units with corresponding Snellen equivalents listed above the axis. (**D**) Survival analysis showing the probability of the least affected eye retaining better than Snellen 20/400 in patients with p.(Gly1961Glu) (blue curve) or with rare and frequent hypomorphic alleles (green curve) and all other patients (black curves). Color-matched dotted lines represent 95% confidence intervals for each individual curve.

**Figure 3:**
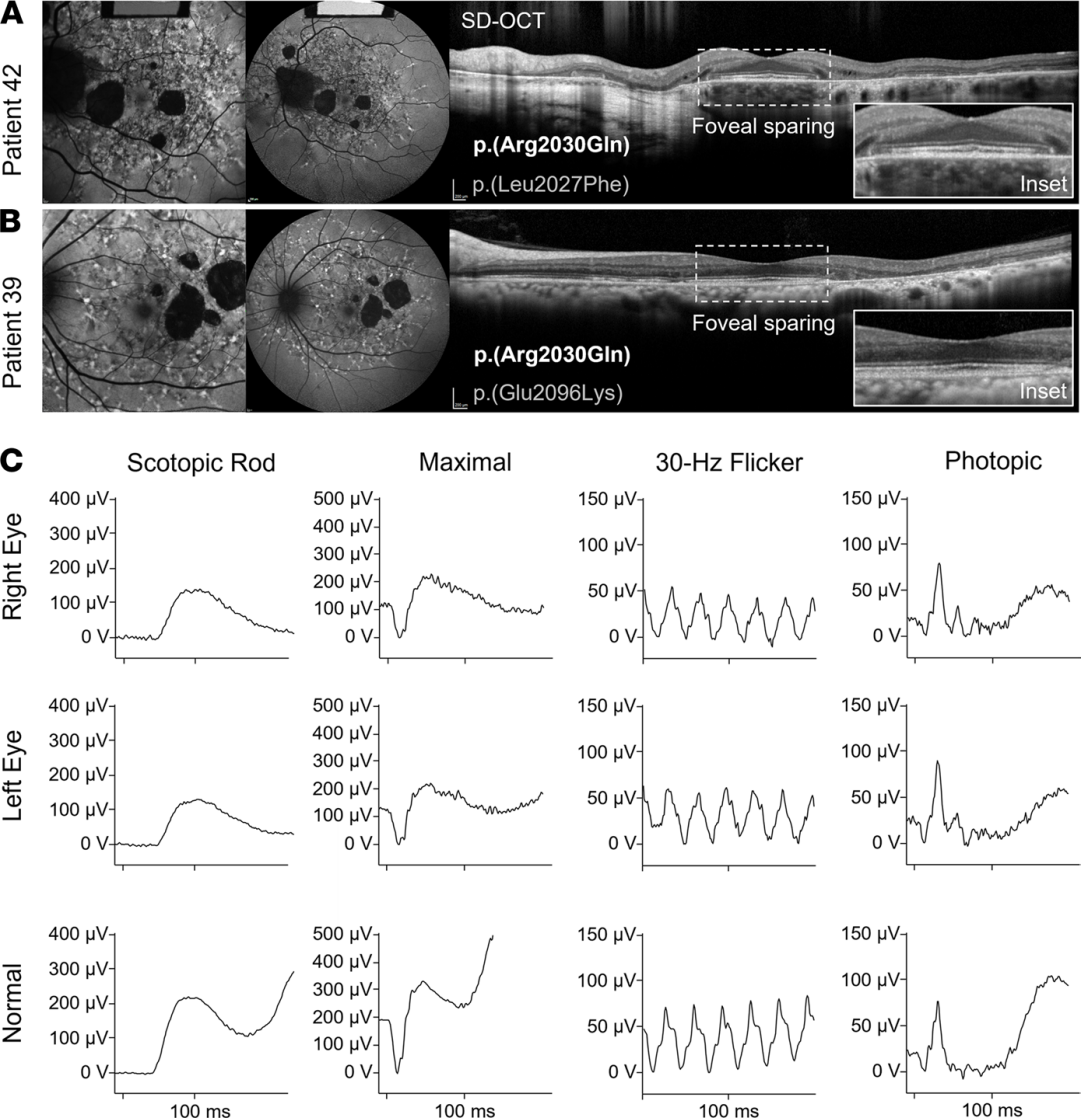
Retinal phenotype of the rare hypomorph p.(Arg2030Gln) variant of ABCA4 disease. Macular 30° autofluorescence and 55° autofluorescence images and horizontal (9 mm) SD-OCT scans of the (**A**) left eye of patient 42 and (**B**) left eye of patient 40. SD-OCT scans with enlarged insets (2 mm) of the fovea show preservation of outer retinal layers resulting in 20/20 vision in the eyes of both patients. (**C**) Unimpaired full-field scotopic (dark-adapted 0.01 rod), maximal (dark-adapted 3.0 combined rod and cone), 30 Hz flicker, and photopic (light-adapted 3.0 single flash cone) electroretinogram responses of the right and left eyes of patient 39 and representative waveforms from an age-matched healthy control eye.

**Figure 4:**
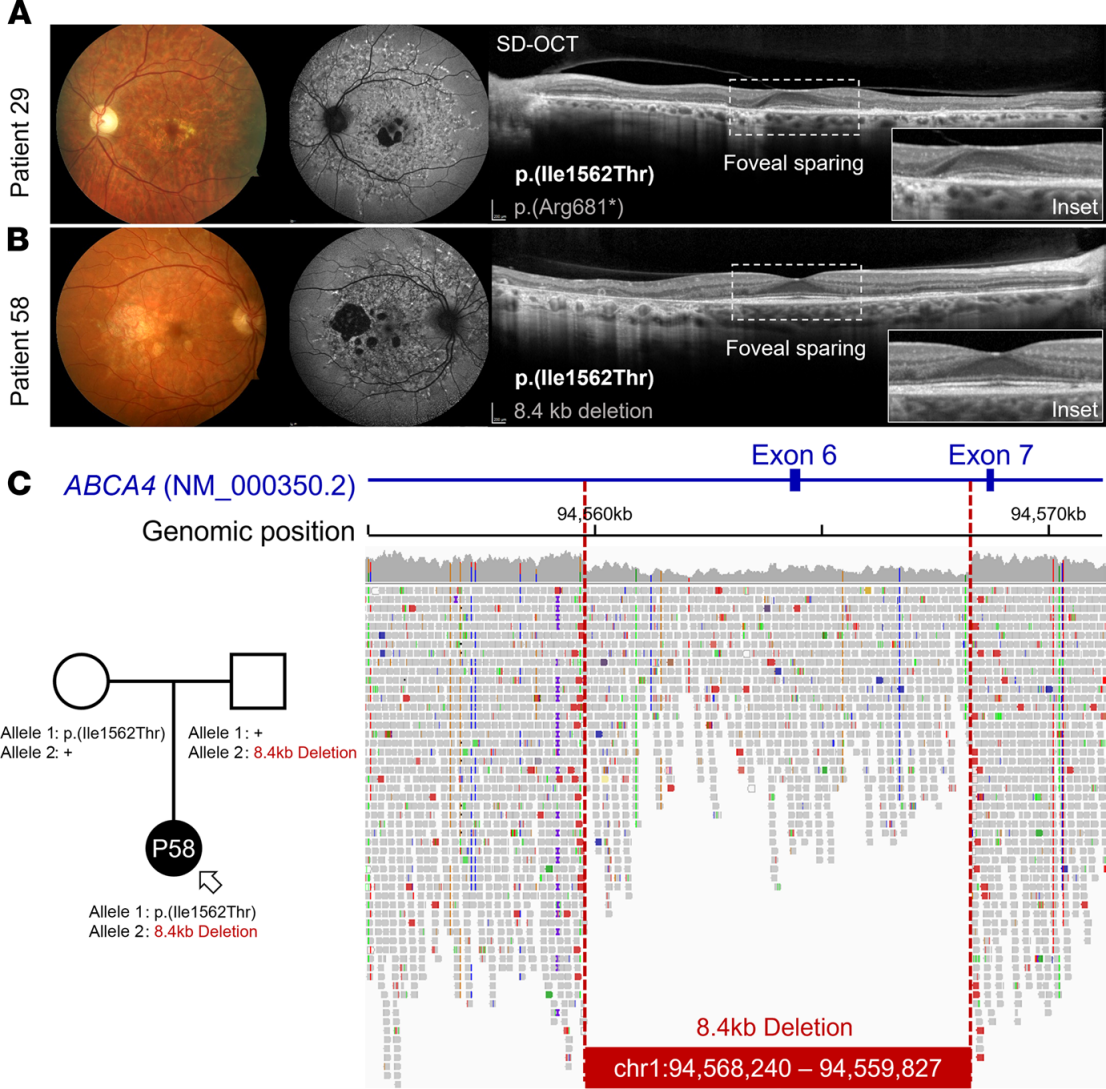
Retinal phenotype of the rare hypomorph p.(Ile1562Thr) variant of ABCA4 disease. Color fundus photographs, autofluorescence images, and horizontal (9 mm) SD-OCT of the (**A**) left eye of patient 29 and (**B**) right eye of patient 58. SD-OCT scans with enlarged insets (2 mm) of the fovea show preservation of outer retinal layers resulting in unimpaired 20/20 vision in the eyes of both patients. (**C**) Pedigree showing segregation of the p.(Ile1562Thr) and large 8.4 kb deletion alleles in patient 58. Pileup of whole-genome sequencing reads showing the approximate size and genomic position of the *ABCA4* deletion, which spans the entire length of exon 6.

Genotypes consisting of the major disease-causing allele, p.(Gly1961Glu), and the frequent hypomorphic allele, p.(Asn1868Ile), were the most prominent variants among the mild phenotypes, together accounting for 56% of patients with prognosis 1 and prognosis 2 (Figure 2A). Despite the advanced age of this cohort, 3 patients (patients 12, 18, and 20) presented with early-stage bull’s eye maculopathy (Supplemental Figure 1, A–C). As we have previously shown, p.(Asn1868Ile) is highly associated with foveal sparing, which is a major contributing factor to the delayed symptom onset age in most patients (Figure 2B and ref. 19). Among the remaining patients in the mild prognosis categories, we identified another group of patients with 6 recurring alleles, p.(Ala1038Val), c.4253+43G>A (18), p.(Pro1486Leu), p.(Thr1526Met), p.(Ile1562Thr), and p.(Arg2030Gln), that have features in common with p.(Asn1868Ile), most notably, delayed symptom onset due to foveal sparing (Table 1 and Figure 2, C and D). Disease features in the fundus of these cases were confined to a definable area around the vascular arcades in a reticular appearance studded along the peripheral boundary with elongated “tails” projecting eccentrically in a radial pattern (Figure 3, A and B; and Figure 4, A and B). Generalized dysfunction of the cone and rod systems was not detected on ffERG testing (Figure 3C). Although each of these variants is exceedingly rare in the general population (0.005 > MAF > 0.00005), unaffected homozygotes have been reported for p.(Ala1038Val), p.(Ile1562Thr), and c.4253+43G>A, resulting in some cases in conflicting interpretations of pathogenicity (Supplemental Table 2). Furthermore, as has been observed with p.(Asn1868Ile), the alleles in *trans* in these genotypes are mostly loss-of-function alleles, including an 8.4 kb deletion identified in patient 58 (Figure 4C). Considering these differences, we separated these mild *ABCA4* alleles into 3 classes: p.(Gly1961Glu), frequent hypomorph, and rare hypomorph.

### Classification of PVS1 and severe non-PVS1 alleles.

The distribution of PVS1 (i.e., null or loss-of-function alleles) (Table 1) was skewed toward the most severe clinical phenotypes though at a lower-than-expected proportion. Genotypes with a PVS1 allele comprised about 33.3% of prognosis 4 cases while the remaining approximately 66.7% consisted mostly of missense variants and, in part, functionally validated deep intronic and synonymous variants. The majority of these missense alleles have been observed to be the causal allele in *trans* from p.(Asn1868Ile) (19). Using our current data set, we further classified 5 additional alleles, p.(Thr1019Met), p.(Ala1598Asp), p.([Asp1532Asn;Asn1868Ile]), p.([Gly863Ala;Asn1868Ile]), and c.5714+5G>A, as severe based on their recurrence in compound heterozygous and/or homozygous patients with prognosis 3 or prognosis 4. To distinguish these severe non-PVS1 alleles from moderate/milder alleles, we grouped them into a separate “severe” subclass (Table 1).

### Classification of moderate variants.

**Figure 5:**
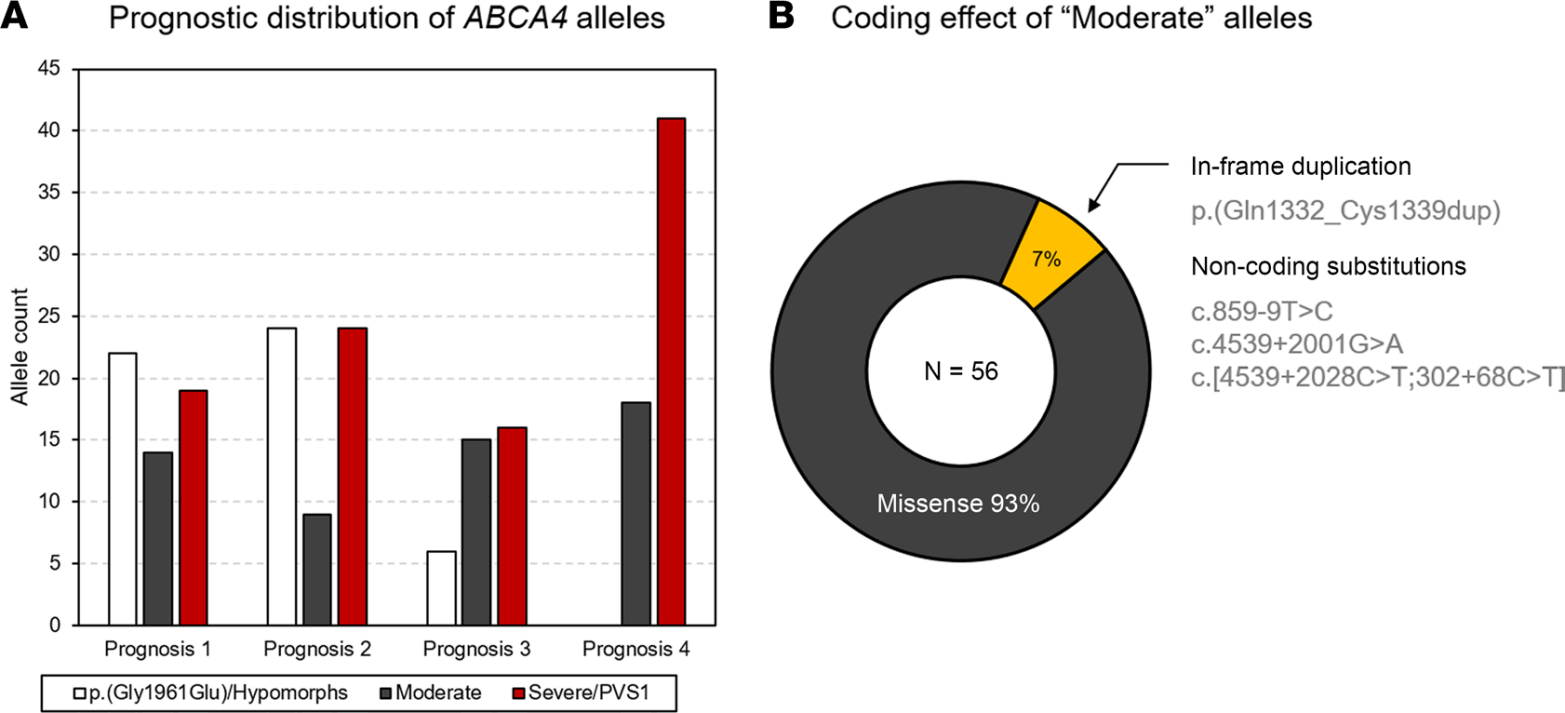
Clinical and genetic characteristics of moderate ABCA4 alleles in the study. (**A**) Distribution of p.(Gly1961Glu) and hypomorphs (white bars) and moderate (gray bars) and severe/PVS1 (red bars) alleles across prognosis categories. (**B**) Coding effect of alleles designated as moderate in patients with ABCA4 disease.

After classifying 65% of alleles in the study cohort as either mild or severe, a remaining group of 36 unique variants (35%, 56 total alleles) did not meet any of the aforementioned classification criteria. These alleles were uniformly distributed across prognosis categories as compared with the other classified allele groups, which skewed accordingly toward mild or severe prognoses (Figure 5A). The coding effect in 93% of these alleles was missense (Figure 5B). The 3 non-missense variants in this group were an exonic in-frame duplication, a deep intronic 15-nucleotide deletion, and the known c.859-9T>C variant, which prior midi-gene studies in HEK293T cells have determined to have a moderate effect as the variant results in 75% of wild-type *ABCA4* RNA (16). Considering the nonspecific genetic attributes of these alleles and their collectively uniform distribution across prognosis categories, we classified them in a moderate group.

### Construction of a genotype-phenotype correlation matrix.

**Figure 6:**
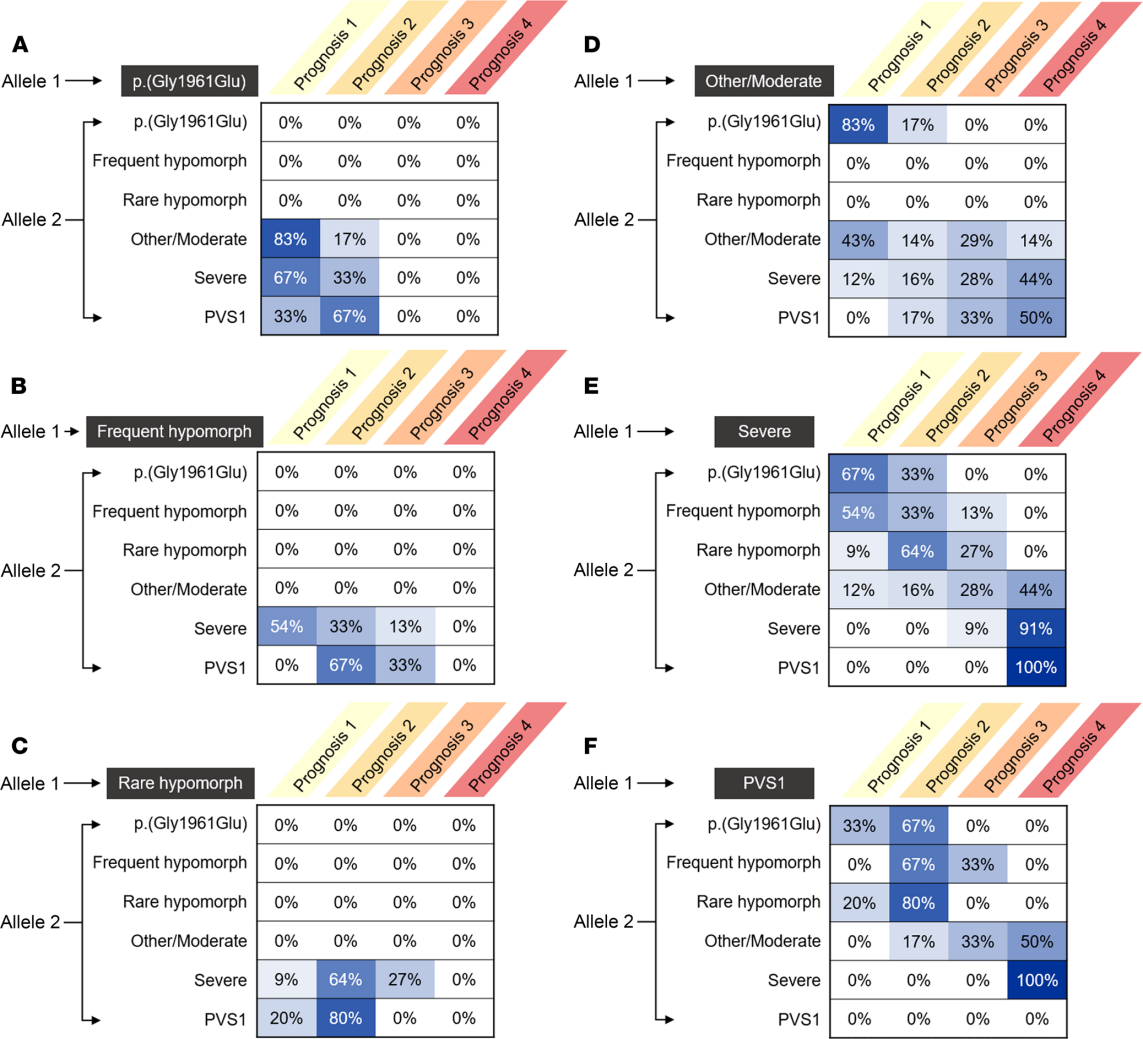
Prognostic probabilities (%) of all possible combinations for each allele class. (**A**) p.(Gly1961Glu), (**B**) frequent hypomorph, (**C**) rare hypomorph, (**D**) moderate, (**E**) severe and (**F**) PVS1. Percentages represent the observed fraction of patients across each prognosis category for a given allele 1 and allele 2 combination.

We generated probability matrices representing correlations among the 4 clinical prognosis categories (prognoses 1–4) and all possible genotypic combinations for the following allele classes: p.(Gly1961Glu), frequent hypomorph, rare hypomorph, moderate, severe, and PVS1 (Table 1 and Figure 6). Genotypes consisting of a p.(Gly1961Glu), frequent hypomorphic, or rare hypomorphic allele had the mildest prognostic outcomes, with most cases having either prognosis 1 or prognosis 2 (Figure 6, A–C). Genotypes of these 3 allele classes were also the least heterogenous in terms of prognostic distribution (*P* = 0.1164, 2-sided Fisher’s exact test [FET], see Supplemental Data 4) compared with both moderate and severe/PVS1 genotypes (*P* < 0.001, 2-sided FET, see Supplemental Data 4). This is due at least in part to the absence of homozygotes and cases with other mild allele combinations. The apparent nonpenetrance, coupled with the consistent clinical phenotype, suggests that these 3 allele classes exhibit a form of “clinical dominance” whereby the allele in *trans*, while necessary for disease expression, has minimal to no effect on the phenotypic variability.

**Figure 7:**
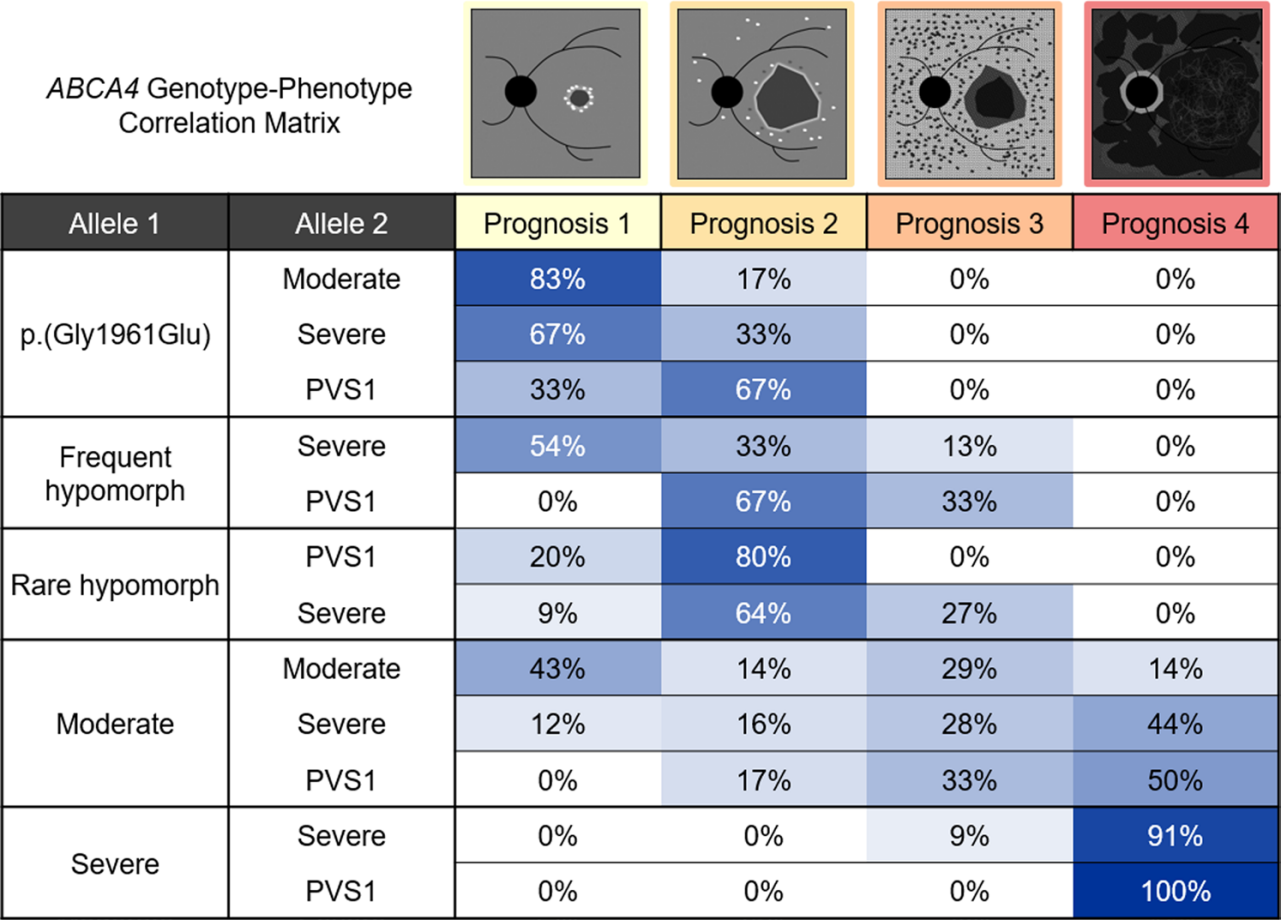
Genotype-phenotype correlation matrix based on the long-term prognostic outcomes of 112 genetically confirmed patients with ABCA4 disease. Percentages represent the observed fraction of patients across each prognosis category for a given allele 1 and allele 2 combination. For the list of unclassified variants, see Table 1 or Supplemental Table 2.

Conversely, all prognosis categories were represented in moderate, severe, and PVS1 allele combinations, and the additive severity of the allele in *trans* strongly correlated with the prognostic severity for these allele combinations. For instance, moderate allele genotypes with another moderate allele in *trans* gave a 43% probability of having prognosis 1 whereas having a p.(Gly1961Glu) allele in *trans* increased the prognosis 1 probability to 83%, and having a severe or PVS1 allele in *trans* reduced the prognosis 1 probability to 0% to 12% and increased the probability of prognoses 3 and 4 to 44% to 55% (Figure 6D). Similar trends appeared for both severe and PVS1 genotypes. Prognosis correlations between PVS1 and severe allele genotypes were also remarkably similar, suggesting very little clinical distinction between the 2 allele classes (Figure 6, E and F). To simplify these observations for clinical applicability, we excluded allele combinations that were not present in patients for any prognosis, thereby collapsing each allele matrix into only those representing the genotypes of all patients across the study cohort (Figure 7).

## Discussion

Advances in genomic medicine in recent decades have allowed genetic testing in the clinic to be a routine option for patients with monogenic diseases. While this has undoubtedly improved the standard of care for patients, the utility of a genetic result rarely extends beyond diagnostic confirmation. The underleveraging of variant level insight in the clinic is attributable to the lack of concrete genotype-phenotype correlations, which are difficult to assess for several reasons. First, Mendelian disorders like ABCA4 disease are both rare and profoundly heterogeneous. Prior studies have noted strong trends with specific alleles (12, 20–24); however, most cohorts are typically insufficient in size and scope to make conclusions that are applicable to clinical care. Moreover, cross-sectional study cohorts themselves are demographically heterogeneous, particularly in terms of age, adding further limitations, such as unknown disease trajectory and clinical outcome of younger patients.

The large clinical and genetic repository we have built over 20+ years has allowed us to overcome most of these issues. Using the well-characterized clinical data of an age-restricted (≥50 years of age) cohort of 112 patients, we were able to precisely dissect apart the complex genotype-phenotype correlation landscape of ABCA4 disease in a quantitative manner that can be immediately used to assess and predict the long-term prognosis of patients following genetic testing. The correlation matrix can be improved upon by the addition of more cases in follow-up studies to increase statistical power and accommodate other *ABCA4* variants not described in this study. These data also provide precise insight into magnitude differences in disease severity between different alleles, which should be considered in the selection of patients for clinical trials.

These data can also be used to clinically classify the pathogenicity of different *ABCA4* alleles. Analyzing patients with the mildest prognoses, for instance, identified a class of rare hypomorphic variants that exhibit clinical overlap with p.(Asn1868Ile) cases, including slowly progressing disease and persistent sparing of the fovea. Results of prior functional and clinical studies of these variants were also consistent with mild characterization. For instance, transgenic expression of human p.(Ala1038Val) in both *Xenopus* *laevis* tadpole retinae and HEK293T cells revealed no observable defects in subcellular localization (25, 26). The latter study also showed that p.(Ala1038Val) mutant structure closely resembles the wild-type ABCA4 structure using single-particle analysis (cryo-electron microscopy; ref. 26). The clinical phenotypes of all well-characterized patients harboring p.(Ala1038Val) (26), p.(Arg2030Gln) (27, 28), p.(Pro1486Leu) (29), p.(Thre1526Met) (27, 28, 30), and p.(Ile1562Thr) (30, 31) alleles in the literature are also consistent with milder disease in general and with specific hypomorphic features.

These and other mild *ABCA4* alleles, including p.(Gly1961Glu) and the frequent hypomorph p.(Asn1868Ile), also exhibit some collective characteristics that are inconsistent with most autosomal-recessive diseases. Under an additive pathogenicity model that has been proposed for ABCA4 (32), patient phenotypes are expected to vary according to the combined effects of both *ABCA4* alleles, and indeed, the phenotypic outcomes of moderate and severe/PVS1 alleles vary widely depending on the allele in *trans* (Figure 5, D–F). Mild alleles, however, appear to be “clinically dominant” in that all genotypes are invariably mild in overall severity (long-term prognosis), and additionally, each respective allele has unique and consistent subphenotypic features (e.g., foveal sparing [hypomorphs] and optical gap [p.(Gly1961Glu)]) irrespective of the type of allele in *trans*. This phenomenon may be partially explained by the nonpenetrance of mild genotypes resulting in more “homogeneous” genotype combinations in observed cases. The underlying mechanisms resulting in subphenotypes, while of diagnostic value for solving cases without genetic confirmation, remain unknown.

Our analysis also reclassified several non-PVS1 alleles as clinically severe, such as the c.5714+5G>A substitution in intron 40, which was previously reported to have a “moderate” effect as it results in approximately 39% correctly spliced mRNA in HEK293T cells (16). Consistent with other clinical studies (6, 28, 33, 34), we also found the variant to be exclusively associated with severe phenotypes (compound heterozygous in 2 patients with prognosis 3 and 3 patients with prognosis 4), which led us to the conclude that the allele is at least clinically severe in patients. Several other missense alleles were also classified as severe, including p.(Ala1598Asp), p.(Thr1019Met), and p.([Asp1532Asn;Asn1868Ile]), based on their recurrence in patients (including homozygotes) with prognoses 3 and 4. These, and a large group of other missense alleles, comprised an unexpectedly large proportion of genotypes leading to the most severe prognostic outcomes. This observation should caution against the common interpretation that most missense variants, at least in the *ABCA4* gene, are less severe than PVS1 variants.

This study has several limitations. While the patient cohort is large considering the rarity of this disease, not all possible ABCA4 genotypes are represented. Notably, biallelic PVS1 genotypes, which are known to underlie the most severe ABCA4 disease phenotypes, such as retinitis pigmentosa like, ROC, and cone-rod dystrophy, were not included (refs. 6–10). Prognostic assessment in these cases, however, is usually unambiguous as visual deterioration begins early in life and the disease progresses rapidly. The 4 prognostic classifications defined in the study may also not fully represent the breadth of clinical outcomes in ABCA4 disease. Further studies based on our study design in larger, preferably multiethnic, cohorts of more comprehensively characterized patients would help address many of the current limitations and critically advance precision medicine for ABCA4 disease. In summary, we constructed a genotype-phenotype matrix based on the long-term prognostic outcomes of 112 genetically confirmed patients with ABCA4 disease. Two major disease-causing variants of ABCA4, p.(Gly1961Glu) and p.(Asn1868Ile), accounted for more than half of the genotypes (patients) with the mildest prognoses. We also identified a potentially new class of rare hypomorphic variants among cases with mild prognoses, which, together with p.(Gly1961Glu) and p.(Asn1868Ile), exhibit “clinical dominance” in their consistent clinical features irrespective of the allele in *trans*. We identified a large group of missense variants that are associated with the more severe prognoses and clinically reclassified others, including c.5714+5G>A, that were previously suggested to be nonsevere. The genotype-phenotype correlation matrix provides prognostic probabilities based on underlying *ABCA4* genotype and can be used as a tool to assess disease severity in patients and as a framework for design of and selection of patients for clinical trials.

## Methods

### Study participants and clinical characterization.

Patients diagnosed with Stargardt or *ABCA4*-related disease were recruited from the Department of Ophthalmology at Columbia University Irving Medical Center. In total, 112 unrelated patients harboring 2 pathogenic variants in *ABCA4* and ≥50 years of age were included in the study. The lower age limit threshold of 50 years was chosen to ensure that all major genotype groups were accommodated in the analysis, particularly patients with the common hypomorphic allele, p.(Asn1868Ile), whose median age of symptom onset is approximately 35 years (IQR 28–48 years; ref. 19). Each patient underwent a complete ophthalmic examination by a retinal physician, which included slit-lamp and dilated fundus examination, BCVA (Snellen), color fundus photography, fundus autofluorescence (AF, 488 mn, 532 nm, and 787 nm), SD-OCT scanning, and ffERG testing. Conversion of CF and HM to logMAR units was calculated in accordance with Schulze-Bonsel et al. (35). In short, CF was replaced with the calculated decimal acuity of 0.014, which corresponds to approximately Snellen 20/1500 or logMAR 1.875; HM was replaced with the decimal acuity of 0.005, which corresponds to approximately Snellen 20/4000 or logMAR 2.300.

Imaging across all modalities was conducted following pupil dilation (>7 mm) with tropicamide (1%) and phenylephrine hydrochloride (2.5%). Fundus AF (488 nm) images and 9 mm horizontal foveal SD-OCT scans were acquired with the Spectralis HRA+OCT (Heidelberg Engineering). Ultra-wide-field AF images were acquired with an Optos 200 Tx (Optos PLC). ffERGs were recorded on silver-impregnated fiber electrodes (DTL, Diagnosys LLC) on the Espion Visual Electrophysiology System (Diagnosys LLC) in accordance with International Society for Clinical Electrophysiology of Vision (ISCEV) standards (36). ffERG classifications were assigned according to electrophysiological attributes described by Lois et al. (23, 37). Group 1 is characterized by no detectable loss in scotopic or photopic function; group 2 is characterized by photopic loss but normal scotopic function; and group 3 exhibits deterioration of both scotopic and photopic function.

Prognosis classifications (prognoses 1, 2, 3, or 4) were determined by 2 independent graders using 55° AF (488 nm) images of each eye for all study patients. In patients with interocular discordance, the prognosis classification was assigned according to the more advanced eye. Discordant evaluations between graders were adjudicated by an additional grader. Notes from the corresponding clinical exam, which included direct and indirect ophthalmoscopy details, were reviewed to confirm the final prognosis group assignment in each patient. All 3 graders were blinded to the *ABCA4* genotype of each patient at the time of prognosis classification.

### Molecular analyses.

Screening of the *ABCA4* gene was performed by next-generation sequencing as previously described (38, 39). All detected possibly disease-associated variants were confirmed by Sanger sequencing and analyzed with Alamut software (Interactive Biosoftware). Segregation of the new variants with the disease was analyzed in families if family members were available. Functional annotation of variants was determined using computational software including ANNOVAR (40) using pathogenicity scores of M-CAP, REVEL, Eigen, and CADD (v1.6). As a general guideline, pathogenic consequences are predicted for variants with scores over 0.025 for M-CAP, 0.5 for REVEL, 0.5 for Eigen, and 20 for CADD. The allele frequencies of all variants were compared to those in the Genome Aggregation Database (accessed October 2021).

### Statistics.

A detailed summary of all statistical calculations is provided in Supplemental Data 1–4. Comparison of mean characteristics between prognosis categories was determined by a 1-way ANOVA test with post hoc Tukey’s honestly significant differences and Kruskal-Wallis tests. Significance was set at α level less than 0.05. Density plots were generated using the ggridges package in R version 4.0.4. FETs for count data (2 × 3 contingency table) were used to compare the distributions of mild, moderate, and severe allele combinations across prognosis categories.

### Study approval.

All study procedures were defined under protocol AAAI9906 approved by the Institutional Review Board at Columbia University Irving Medical Center. The study adhered to tenets set out in the Declaration of Helsinki.

## Author contributions

WL designed the study, recruited study participants, acquired and analyzed clinical data, and wrote the manuscript; JZ performed sequencing, analyzed molecular data, and critically revised the manuscript; PYS recruited participants and acquired clinical data; TN assisted with molecular analyses; SHT clinically examined study participants; and RA supervised the study, critically revised the manuscript, and obtained research funding.

## Supplementary Material

Supplemental data

ICMJE disclosure forms

Supplemental table 1

Supplemental table 2

## Figures and Tables

**Table 1:**
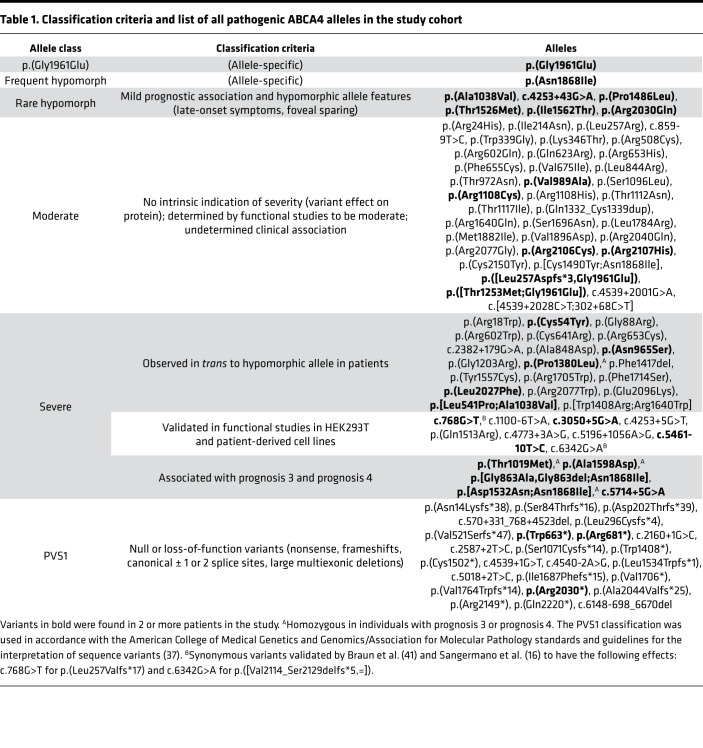
Classification criteria and list of all pathogenic ABCA4 alleles in the study cohort

## Version 1 12/07/2021

In-Press Preview

## Version 2 01/25/2022

Electronic publication
