# Supplementary material for: A genotype-phenotype correlation matrix for ABCA4 disease based on long-term prognostic outcomes
Source: JCI Insight. 2022 Jan 25;7(2):e156154. doi: 10.1172/jci.insight.156154 (PMC8855796; doi:10.1172/jci.insight.156154)
Supplement: Supplemental data [file jciinsight-7-156154-s171.pdf]

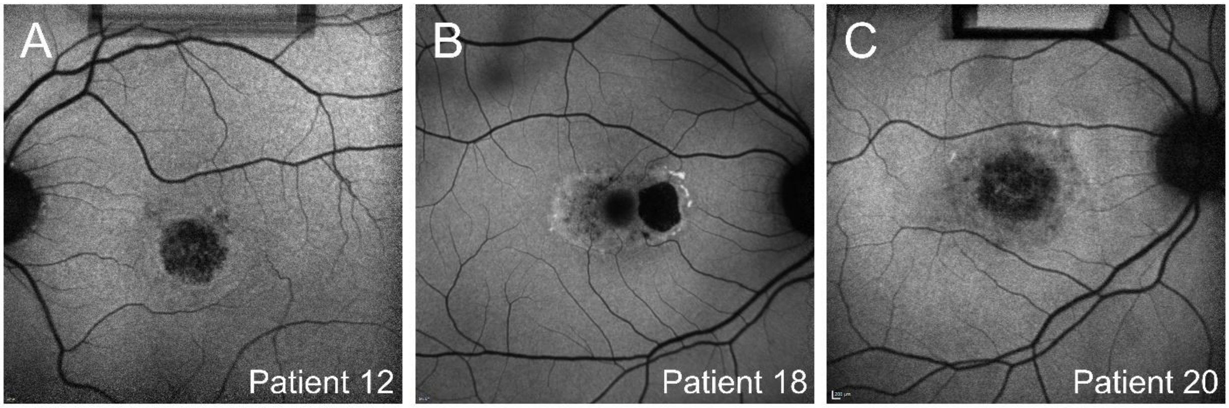

p.(Gly1961Glu) | p.(Asn1868Ile) | p.(Gly1961Glu)
p.([Leu257Aspfs*3,Gly1961Glu]) | p.(Pro1380Leu) | p.(Pro1380Leu)

Supplemental Figure 1: Bull's eye maculopathy (BEM) presentation in advanced age patients with ABCA4 disease. Fundus autofluorescence imaging of the left eye of patient 12 (age 67 years), right eye of patient 18 (age 54 years) and right eye of patients 20 (age 70 years) exhibiting uniform BEM lesions without peripheral flecks. ABCA4 variants for each patients are listed below.

Supplemental Data 1. Summary statistics and comparison of age (years) at examination between patients in Prognosis categories of ABCA4 disease.

Summary Statistics

Prognosis	Ntrial	Mean	SD	Median	MAD	IQR	Trimean
1	28	57.9	7.6	55.0	6.9	14.2	56.6
2	31	61.4	9.7	60.3	12.0	16.3	60.5
3	20	59.1	8.4	56.1	8.3	13.6	57.9
4	33	61.8	8.6	60.0	10.7	13.9	61.0

Abbreviations: SD, standard deviation; MAD, median absolute deviation; IQR, interquartile range.

One-Way ANOVA Test between Prognosis 1, 2, 3 and 4

F-ratio	*P-value*
1.37478	0.254411

Kruskal-Wallis Test between Prognosis 1, 2, 3 and 4

H-statistic	*P-value*
4.4582	0.21605

Post Hoc Tukey HSD

Pair-wise comparisons	*P-value*
Prognosis 1 : Prognosis 2	0.43374
Prognosis 1 : Prognosis 3	0.95585
Prognosis 1 : Prognosis 4	0.34532
Prognosis 2 : Prognosis 3	0.75007
Prognosis 2 : Prognosis 4	0.99863
Prognosis 3 : Prognosis 4	0.65598

Supplemental Data 2. Summary statistics and comparison of age of symptomatic onset between patients in Prognosis categories of ABCA4 disease.

Summary Statistics

Prognosis	Ntrial	Mean	SD	Median	MAD	IQR	Trimean
1	24	41.2	11.4	44.5	8.2	14.2	43.3
2	29	40.9	12.1	45.0	10.4	17.0	42.2
3	17	31.5	17.6	30.0	23.7	33.0	30.8
4	32	17.1	9.6	17.0	10.4	10.2	16.1

Abbreviations: SD, standard deviation; MAD, median absolute deviation; IQR, interquartile range.

One-Way ANOVA Test between Prognosis 1, 2, 3 and 4

F-ratio	*P-value*
25.0399	< 0.00001

Kruskal-Wallis Test between Prognosis 1, 2, 3 and 4

H-statistic	*P-value*
43.1082	< 0.00001

Post Hoc Tukey HSD

Pair-wise comparisons	*P-value*
Prognosis 1 : Prognosis 2	0.99989
Prognosis 1 : Prognosis 3	**0.03759**
Prognosis 1 : Prognosis 4	**<0.0001**
Prognosis 2 : Prognosis 3	**0.04461**
Prognosis 2 : Prognosis 4	**<0.0001**
Prognosis 3 : Prognosis 4	**0.00061**

Statistically significant (P<0.05) values in **bold**

Supplemental Data 3. Summary statistics and comparison of best-corrected visual acuity (logMAR equivalent) of the "better eye" between patients in Prognosis categories of ABCA4 disease.

Summary Statistics

Prognosis	Ntrial	Mean	SD	Median	MAD	IQR	Trimean
1	26	0.4	0.4	0.5	0.6	0.8	0.4
2	31	0.6	0.5	0.6	0.6	0.9	0.6
3	18	0.7	0.7	0.5	0.6	1.0	0.6
4	33	1.6	0.6	1.3	0.4	0.7	1.5

Abbreviations: SD, standard deviation; MAD, median absolute deviation; IQR, interquartile range.

One-Way ANOVA Test between Prognosis 1, 2, 3 and 4

F-ratio	*P-value*
26.94054	< 0.00001

Kruskal-Wallis Test between Prognosis 1, 2, 3 and 4

H-statistic	*P-value*
46.847	< 0.00001

Post Hoc Tukey HSD

Pair-wise comparisons	*P-value*
Prognosis 1 : Prognosis 2	0.55288
Prognosis 1 : Prognosis 3	0.40099
Prognosis 1 : Prognosis 4	**<0.0001**
Prognosis 2 : Prognosis 3	0.99452
Prognosis 2 : Prognosis 4	**<0.0001**
Prognosis 3 : Prognosis 4	**<0.0001**

Statistically significant (P<0.05) values in **bold**

Supplemental Data 4. 2x3 Fisher Exact Test for count data comparing distributions of mild, moderate and severe allele combinations across Prognosis categories.

Mild allele combination cases

**Allele 1**	Mild	**Prognosis 1**	**Prognosis 2**	**Prognosis 3**	**Prognosis 4**
**Allele 2**	Mild	0	0	0	0
	Moderate	5	1	0	0
	Severe	17	23	6	0

Two-sided P-value	*Two-sided P-value (Monte Carlo simulation based on 200 replicates)*
0.1177	0.1164

Moderate allele combination cases

**Allele 1**	Moderate	**Prognosis 1**	**Prognosis 2**	**Prognosis 3**	**Prognosis 4**
**Allele 2**	Mild	5	1	0	0
	Moderate	3	1	2	1
	Severe	3	6	11	17

Two-sided P-value	*Two-sided P-value (Monte Carlo simulation based on 200 replicates)*
**0.0007353**	**0.001499**

Severe allele combination cases

**Allele 1**	Severe	**Prognosis 1**	**Prognosis 2**	**Prognosis 3**	**Prognosis 4**
**Allele 2**	Mild	17	23	6	0
	Moderate	3	6	11	17
	Severe	0	0	1	15

Two-sided P-value	*Two-sided P-value (Monte Carlo simulation based on 200 replicates)*
-	**0.0004998**
